# Intravascular Targets for Molecular Contrast-Enhanced Ultrasound Imaging

**DOI:** 10.3390/ijms13066679

**Published:** 2012-06-01

**Authors:** Siver A. Moestue, Ingrid S. Gribbestad, Rune Hansen

**Affiliations:** 1 MI Lab, Department of Circulation and Medical Imaging, Norwegian University of Science and Technology (NTNU), Trondheim N-7006, Norway; E-Mail: ingrid.s.gribbestad@ntnu.no; 2 Department of Medical Technology, SINTEF Technology and Society, Trondheim N-7491, Norway; E-Mail: rune.hansen@sintef.no

**Keywords:** ultrasound imaging, targeted contrast agents, angiogenesis, molecular imaging, microbubbles, cancer

## Abstract

Molecular targeting of contrast agents for ultrasound imaging is emerging as a new medical imaging modality. It combines advances in ultrasound technology with principles of molecular imaging, thereby allowing non-invasive assessment of biological processes *in vivo*. Preclinical studies have shown that microbubbles, which provide contrast during ultrasound imaging, can be targeted to specific molecular markers. These microbubbles accumulate in tissue with target (over) expression, thereby significantly increasing the ultrasound signal. This concept offers safe and low-cost imaging with high spatial resolution and sensitivity. It is therefore considered to have great potential in cancer imaging, and early-phase clinical trials are ongoing. In this review, we summarize the current literature on targets that have been successfully imaged in preclinical models using molecularly targeted ultrasound contrast agents. Based on preclinical experience, we discuss the potential clinical utility of targeted microbubbles.

## 1. Introduction

Compared with other medical imaging modalities, ultrasound imaging has several advantages. It does not involve radiation, has high spatial resolution and is generally considered cheap, safe and widely available. The introduction of gas-filled microbubbles as contrast agents for ultrasound imaging has further improved the performance and increased the versatility of ultrasound imaging.

In *in vivo* molecular imaging, targeted probes are used for non-invasive imaging of molecules overexpressed in disease. This principle can be used also in ultrasound imaging. Conjugating microbubbles with specific ligands allows non-invasive imaging of target expression with high sensitivity. However, microbubbles are confined to the intravascular compartment. Their use as molecular contrast agents is therefore restricted to diseases which are directly or indirectly associated with altered phenotype of cells present in the intravascular compartment. Nevertheless, several interesting indications for targeted microbubbles exist. For example in atherosclerosis, subendothelial lesions cause inflammatory changes in the endothelium. In solid tumors, the cancer cells stimulate the endothelium and induce neoangiogenesis, leading to upregulation of several molecules on the endothelial surface.

Over the last decade, molecular contrast-enhanced ultrasound imaging has been established as a useful technique for imaging intravascular angiogenic markers in cancer. Proof-of-concept studies in preclinical models have repeatedly demonstrated that retention of targeted microbubbles, and thus the signal intensity of the ultrasound images, reflects the expression of target biomarkers. Here, we summarize the findings from preclinical studies of molecular ultrasound contrast agents with the intention to describe the potential clinical utility of these agents in ultrasound imaging of tumor vasculature.

## 2. Ultrasound Contrast Agents

**Figure 1:**
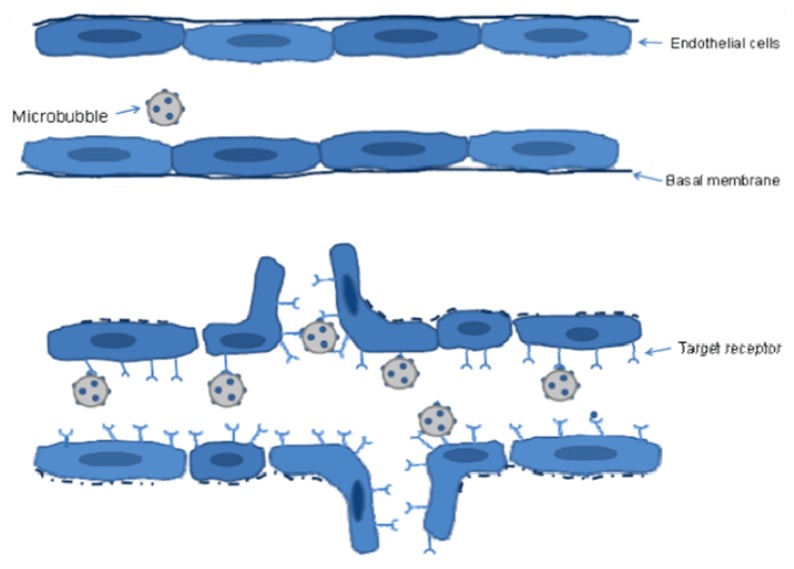
The principle of molecular contrast-enhanced ultrasound. Microbubbles conjugated to specific ligands are injected into the circulation. In healthy capillaries, the expression of target receptors is low. Consequently, the microbubbles do not bind to the target but remain in circulation. In an angiogenic blood vessel, the activated endothelium target receptor is overexpressed. The microbubbles bind to the receptors and accumulate in the vessel. Despite a loss of basal membrane integrity in the diseased vessel, the microbubbles are too large to extravasate and remain in the intravascular compartment.

Contrast agents for ultrasound consist of micrometer-sized gas bubbles stabilized by a thin encapsulating shell made of lipid, albumin or biocompatible polymers which are intravenously administered. Such microbubbles can undergo strong oscillations when exposed to an ultrasound beam. This leads to strong back-scattering of waves enabling detection of the microbubbles and thereby assessment of micro-circulation and perfusion. In the early eighties, Feinstein [1,2] was one of the first to develop air microbubbles encapsulated in protective shells small enough for transpulmonary passage. About 10 years later, the first generation of commercial contrast agents (such as Echovist^®^, Albunex^®^, Levovist^®^) was available. A few microbubble agents with mean diameter of 1–4 microns (Definity^®^, Optison^®^) are currently the only FDA approved contrast agents for ultrasound. The flow pattern of conventional non-targeted microbubbles is typically similar to erythrocytes within the circulation system [3]. Targeting can be achieved through conjugation of disease-specific ligands for the target molecule to the microbubble shell. Molecular imaging with ultrasound relies on detection of targeted microbubbles. Because of the large size of the microbubble, it is only possible to target molecules occurring in plasma or on the surface of the endothelial cells. Seconds after an intravenous bolus administration, contrast agent inflow provides real-time information of blood flow patterns within a region of interest. A few minutes later, imaging of microbubbles attached to molecular targets may be performed and 5–15 minutes after administration most of the free circulating microbubbles are typically cleared from the bloodstream (Figure 1). Differentiating targeted bubbles retained in diseased tissue from free circulating bubbles can be done either by waiting until all circulating bubbles are cleared from the bloodstream or by using a subtraction technique comparing the back-scattered signal before and after a high amplitude destruction pulse has been applied. Free flowing bubbles and bubbles bound to molecular targets at the endothelial cells will potentially have different resonance frequencies due to different oscillation conditions. It is then, for example with the use of dual frequency band detection techniques, possible to differentiate the received echoes from unbound and retained bubbles [4].

### 2.1. Advantages and Disadvantages of Ultrasound Compared to Other Modalities

**Figure 2:**
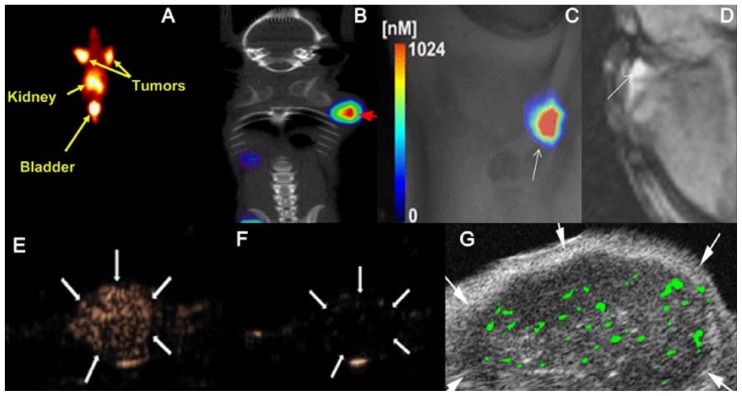
Molecular imaging of the α_v_β_3_ integrin. The advantages and disadvantages of different medical imaging modalities are demonstrated in representative images from tumor-bearing animals. **A**, **B**, **C** and **D** show single photon emission computerized tomography (SPECT), positron emission tomography (PET), optical imaging and magnetic resonance imaging (MRI), respectively. **E** and **F** shows a xenograft tumor after injection of microbubbles conjugated with ariginine-glycine-aspartic acid (RGD) or a scrambled control peptide, respectively. **G** shows the presence of single microbubbles after injection of α_v_β_3_-targeted microbubbles. Reproduced with permission from [9–14].

Ultrasound molecular imaging offers several advantages and some limitations compared to other imaging modalities. Low equipment cost, mobility of equipment and rapid execution of imaging protocols are important advantages of ultrasound. Spatial resolution is comparable to magnetic resonance imaging (MRI) for imaging of relatively small objects close to the transducer (e.g., prostate, breast, and thyroid) [5]. Contrast agent sensitivity is excellent with ultrasound where individual microbubbles can be detected [6]. For MRI, contrast agent sensitivity is a limitation. Computerized tomography (CT), single photon emission computerized tomography (SPECT) and positron emission tomography (PET) have good sensitivity but all of these modalities utilize ionizing radiation and SPECT and PET also suffers from inferior spatial resolution. Another important advantage with ultrasound is the rapid clearance of unbound microbubbles and the possibility to destroy circulating and retained microbubbles within a desired region of interest [7,8]. This allows for repeated examinations and for imaging of multiple molecular targets in the same subject. Finally, ultrasound boasts excellent temporal resolution and allows real-time image evaluation. Regarding disadvantages, a whole-body scan is not feasible with ultrasound and some organs (e.g., lungs and brain without performing a craniotomy) are difficult to examine by ultrasound. When imaging large organs or objects far from the transducer, spatial resolution is typically inferior to MRI and CT. Ultrasound has until recently mainly been a two-dimensional imaging modality but real-time three-dimensional imaging is now starting to be implemented on high-end scanners. In Figure 2, representative images obtained using α_v_β_3_-targeted contrast agents are presented, illustrating differences in sensitivity and spatial resolution between different imaging modalities.

The major limitation of molecular contrast-enhanced ultrasound imaging is that microbubbles are restricted to the vascular lumen, and only targets in this compartment can be imaged.

### 2.2. Requirements for Intravascular Targets in Ultrasound Imaging

Intravascular targets for contrast-enhanced ultrasound imaging must meet the same requirements as other targets for molecular contrast agents. First, the target should be extracellular. These targets in general are easier to image than intracellular targets, as internalization of contrast agent is not required [15]. Second, the target should be expressed in high numbers. By pure stoichiometry, this allows binding of large amounts of contrast agent, which enhances the sensitivity of disease detection. Third, the expression of the target must be altered significantly in order to distinguish between the normal and diseased state of the tissue, at a clinically relevant time point. The magnitude of the altered expression in diseased tissue represents the maximum achievable target-to-background ratio that can be achieved during imaging. The unspecific binding of the contrast agent should be as low as possible in order to reduce background signal. This is typically tested by comparison of targeted microbubbles and microbubbles with isotype (non-binding) control ligand. Combining an intravascular target with low native abundance and high upregulation in disease with a microbubble conjugated to a highly specific ligand is therefore a requirement for successful differentiation between lesions and normal tissue.

Antibodies usually have high binding specificity and are frequently used in target validation or proof-of-concept studies. However, due to manufacturing issues and the risk of immunological adverse effects, antibodies are less clinically desirable [16]. Therefore, the use of low-molecular ligands, such as small peptides, is advantageous. Such ligands are generally easier to manipulate and conjugate with the reporter part of the contrast agent. The binding kinetics may be improved compared to antibodies, and such ligands may be incorporated in the microbubble shell instead of being conjugated using the biotin-streptavidin linker [17,18]. Ideally, the ligand should not exert any pharmacological effect, as this increases the risk of adverse side effects. The binding of ligand to the target should be specific, rapid and strong. The shear forces may rapidly move the contrast agent away from the target if it does not bind with sufficient strength within a short period of time. The effect of shear forces on microbubble binding is less in capillaries, where the blood flow is much lower.

The intravascular confinement of microbubbles represents both a limitation and an advantage. As the bubbles do not extravasate, they can only attach to targets expressed on the luminal side of endothelial cells. This limits the number of possible targets. However, this also allows imaging of the vascular pathology without any interference from extravascular tissue. The selectivity for vascular endothelium may be advantageous in cases where the target receptor is expressed both in the vascular compartment and in the surrounding tissues, or if extensive unspecific binding of ligand is observed in the surrounding tissue.

## 3. Molecular Ultrasound Imaging of Cancer

Cancer can be defined as diseases where cells divide in an uncontrolled manner and are able to invade other tissues. Solid tumors depend on formation of new blood vessels in order to grow beyond a size of 1–2 mm [19,20]. As changes in vascular architecture and function in the affected tissue is an integrated part of the pathology, assessment of blood vessels and their function is a useful approach in imaging of cancer [21,22]. The progression to invasive and eventually metastatic cancer depends on angiogenesis, and imaging of this process is an important approach for early detection and characterization of cancer.

Development of angiogenic capability is typically acquired at a relatively late time point during tumorigenesis [19]. To explain this, the “angiogenic switch” model has been developed [23]. The relative balance between inhibitors and activators of angiogenesis is in general in favor of vascular quiescence. At some point in tumor development, loss of inhibitors or increased amount of activators (for example due to cumulative mutations in oncogenic signaling pathways) turns the balance in favor of angiogenesis, ultimately tipping it in favor of new blood vessel growth. This understanding has led to the development of antiangiogenic drugs, which impair the vascularisation of tumors. Although this in theory should deprive tumors of oxygen and nutrients, the clinical efficacy of antiangiogenic drugs has turned out to be smaller than expected [24].

The tumor vasculature has a different morphology and biology than mature, quiescent vessels [25]. It is tortuous and leaky, and the blood flow is often compromised. The phenotype of the endothelial cells is changed in response to the angiogenic activator molecules, and they overexpress a number of proteins compared to normal endothelium. These proteins are potential targets for drugs or contrast agents. Findings from *in vivo* studies of ultrasound contrast agents targeting angiogenesis are summarized in Table 1.

### 3.1. VEGFR2

The most important molecule for control of the angiogenic process is the vascular endothelial growth factor A (VEGFA), which is produced by cancer cells as a response to hypoxia. VEGFA binds to the VEGF-receptor 2 (VEGFR2) on endothelial cells, activates the cells and induces vascular sprouting. Upregulation of VEGFR2 is observed both in animal models of cancer and in humans, and its expression is a prognostic marker in a variety of malignancies. In gliomas, the expression of VEGFR2 is 3–5 fold higher in tumor vasculature than in normal vasculature [39,40]. Based on the physiological and pathophysiological properties of VEGFR2 it is considered an attractive target for imaging agents for use in all modalities. However, as the number of endothelial cells is far less than the number of tumor cells, high sensitivity is a prerequisite for imaging VEGF receptors. It has repeatedly been demonstrated that molecular contrast-enhanced ultrasound can be used to image VEGFR expression [31,41,42]. The most widely used approach for targeting microbubbles to VEGFR2 is by conjugating anti-VEGFR2 antibodies to the shell. The performance of such microbubbles is similar to that observed using heterodimer-based peptide ligands, with 3 to 4-fold increase in signal intensity in angiosarcoma and glioma models [31]. Although the targeting efficacy may differ, preclinical studies using different VEGFR2-targeting ligands have demonstrated the superiority of targeted versus non-targeted microbubbles and confirmed that VEGFR is a valid target in angiogenesis imaging. The target-to-background ratio achieved using ultrasound imaging is similar to that achieved using SPECT and PET agents [43,44]. However, the achieved target-to-background ratio depends strongly on the VEGFR2 expression and the vascular architecture of the experimental model.

In a recent publication, Pysz *et al*. studied the target specificity of the VEGFR2-targeting microbubble BR55 [26]. This contrast agent is conjugated with a lipopeptide, which is less immunogenic than streptavidin-linked antibodies, improving its clinical translatability. In colon cancer xenografts, more than 3-fold higher signal intensity for BR55 compared to non-targeted control microbubbles was observed. The VEGFR2-targeting microbubbles provided 20-fold higher signal intensity in the xenograft tumors compared to muscle tissue, due to combined effects of increased vascularity and specific targeting of VEGFR2. In a rat prostate cancer model, the same microbubbles had significantly better imaging performance, and a much longer residence time in the tumor tissue, than non-targeted microbubbles [28]. In two different breast cancer xenografts, more than 2.5-fold higher signal intensity was observed in a highly vascularised, aggressive model (MDA-MB-231) compared to a poorly vascularised model (MCF-7) using BR55 [27]. Based on preclinical findings, the BR55 microbubble has entered clinical trials. An early report in 12 prostate cancer patients indicated that this VEGFR2-targeted microbubble improved prostate cancer detection and localization [45].

### 3.2. Integrins

Integrins are a family of cell surface receptors whose primary ligands are extracellular matrix proteins. A full description of integrin biology in cancer is beyond the scope of this report, but has recently been reviewed by Avraamides *et al*. [46]. Unlike quiescent endothelium, tumor-associated endothelium (and sites of wound healing and inflammation) express the integrin receptor α_v_β_3_ [47]. It is believed that the activation of integrin receptors stimulate synthesis of proteolytic enzymes such as matrix metalloproteinases, which may degrade the surrounding extracellular matrix components and create space for formation of a new vessel [48]. In addition, integrin receptors play a role in adhesion of endothelial cells to each other and the exracellular matrix during angiogenesis.

Interestingly, expression of α_v_β_3_ occurs at a later stage in angiogenesis than VEGFR [25]. This may be relevant when considering VEGFR2 and α_v_β_3_ as targets for intravascular contrast agents. These two targets represent cells in different stages of angiogenesis. Although both targets may be expressed at the same time in a tumor, the number of cells expressing each target may vary in different stages of tumor progression. Targeting the α_v_β_3_ receptor has been widely used approach in the development of targeted contrast agents. This is in part due to its biological properties, but also to the fact that the ariginine-glycine-aspartic acid (RGD) amino acid sequence is a well-defined and versatile ligand for this receptor. Cyclic peptide sequences have been found to have greater affinity to the α_v_β_3_ receptor than linear variants [49].

The first preclinical studies of microbubbles targeting the α_v_β_3_-receptor were reported by Leong-Poi *et al*. [50]. Here, microbubbles conjugated to anti-α_v_ antibodies or echistatin showed selective retention in FGF2-induced muscular angiogenesis. It was demonstrated that the retention was caused by attachment to endothelial cells rather than size-dependent entrapment. Later studies confirmed the signal intensity of microbubbles conjugated to echistatin correlated both to integrin expression and tumor blood volume in a rat xenograft model of glioma, demonstrating the potential of noninvasive imaging of tumor angiogenesis [33]. In this study, tumor growth was associated with increased blood volume and increased signal intensity in the ultrasound images, in particular in the peripheral regions of the tumors. This demonstrates how molecular contrast-enhanced ultrasound can be used to study both spatial variation in tumor angiogenesis and changes in vascularity over time. Another approach for targeting α_v_β_3_ overexpression in tumor vasculature is disulfide-constrained cystin knots (knottins). These small peptides have been shown to bind α_v_β_3_ with low nanomolar affinity. Direct comparison in mouse xenograft models indicated that knottin-based and RGD-based microbubbles have higher target-to-background ratio than anti-α_v_β_3_ antibody-based microbubbles [14]. More recently, it has been reported that microbubbles conjugated to a cyclic RGD ligand has high affinity for tumor vasculature *in vivo* [9]. For clinical translation, the use of RGD-based ligands is more desirable than anti-α_v_β_3_-antibodies.

### 3.3. Endoglin

Another intravascular target for imaging of angiogenesis is endoglin (CD105). This is a member of the TGF-β family of receptors, which is required for endothelial cell proliferation. Overexpression of endoglin is associated with poor prognosis in several cancers [51]. Interestingly, CD105 is selectively expressed on angiogenic endothelial cells at significantly higher levels (up to 3 × 10^6^ copies per cell) than other angiogenesis-related targets such as the VEGFRs (<0.2 × 10^6^ copies per cell) [52]. Anti-endoglin antibodies have been conjugated to microbubbles for contrast-enhanced ultrasound imaging. Initial studies demonstrated affinity for endothelial cells *in vitro* [53]. These studies were followed up by studies in mice carrying pancreatic cancer xenografts, demonstrating approximately 10-fold higher signal intensity in tumor tissue than surrounding tissue [30]. Endoglin-targeting and VEGFR2-targeting microbubbles showed comparable signal intensities which might be due to similar marker expression levels in the tumors.

### 3.4. Prostate-Specific Membrane Antigen

Prostate-specific membrane antigen (PSMA) is predominantly localized to the epithelial cells of the prostate gland, but its function is not fully understood. It is upregulated several-fold in high-grade prostate cancers [54]. Interestingly, PSMA is also upregulated on the surface of tumor endothelium, not only in prostate cancer but in other cancers as well [55]. It is therefore a potential target for ultrasound contrast agents. Antibodies targeting the extracellular domain of PSMA have been developed, and when linked to PET nuclides these show high tumor-to-background ratio both in preclinical and clinical studies [56]. More recently, glutamate-urea-lysine analogues have been developed as inhibitors of PSMA. These peptide ligands show high selectivity and high tumor-to-background ratio in xenograft models when used in SPECT imaging [57,58]. A recent paper has described the development of prototype microbubbles targeting PSMA using a glutamate-urea-lysine analogue. These were shown to bind to prostate cancer cells *in vitro* [54]. However, no *in vivo* studies of PMSA-targeting microbubbles have been reported and the potential for successful ultrasound imaging of vascular PSMA is therefore currently unknown.

### 3.5. Inflammatory Markers

Imaging of conditions associated with mild or chronic inflammation has been performed using contrast agents decorated with antibodies or other ligands to endothelial cell adhesion molecules. Since cancer frequently is associated with inflammation, ultrasound contrast agents targeting these proteins may potentially also be of value in cancer imaging. This has been demonstrated in the case of Intercellular Adhesion Molecule 1 (ICAM-1), which is associated with activated endothelial cells, promoting the arrest of leukocytes to inflammatory foci [59]. In a subcutaneous prostate cancer model in rats, specific accumulation of microbubbles targeting ICAM-1 was similar to that of α_v_β_3_ targeted microbubbles [36]. Although the aim of this study was to demonstrate how two different targeted microbubbles can report on treatment-associated changes in tumor biology in the same imaging session, the results also indicate that markers for vascular inflammation can be relevant in molecular ultrasound imaging of cancer. Using triple-targeting microbubbles, it has also been shown that targeting the adhesion molecule, *P*-selectin, further improves the binding efficacy of VEGFR- and α_v_β_3_-targeted microbubbles [38]. Microbubbles targeting adhesion molecules could therefore potentially be useful tools for assessing the inflammatory component of solid tumors.

## 4. Therapy Monitoring in Cancer Using Molecular Contrast-Enhanced Ultrasound Imaging

The principle of personalized medicine in cancer has led to an increased need for methods that can detect response to treatment, including antivascular and antiangiogenic drugs. Identification of responders to targeted anticancer drugs will be increasingly important as more drugs reach clinical practice. Ultrasound imaging is a suitable modality for this purpose, since it is a non-invasive, portable and non-radiative modality. In addition, it can be used to assess vascular function at several levels, including perfusion, blood volume and the expression of endothelial vascular markers [60,61]. Several studies have demonstrated that targeted microbubbles can be used for non-invasive assessment of angiogenic markers. Korpanty *et al*. [30] demonstrated that accumulation of both endoglin- and VEGF-targeting microbubbles correlated with target expression and microvessel density (MVD) in pancreatic xenograft tumors. Treatment with anti-VEGF antibodies detectably reduced the binding of microbubbles to endothelial cells. Similar findings have been reported using the VEGFR-targeting BR55 microbubble in colon cancer xenografts, where both VEGFR expression, MVD and imaging signal was significantly reduced after anti-VEGF antibody treatment [26]. Using an α_v_β_3_-targeted contrast agent, molecular ultrasound imaging has been shown to discriminate between responding and non-responding xenograft tumors [62]. Following bevacizumab treatment, decrease in α_v_β_3_-expressing vasculature was paralleled by a relative reduction in tumor blood volume in the responding tumor model only. In these experiments, adhesion of targeted microbubbles was a more consistent marker for response to treatment than the relative blood volume. Combining several vascular parameters obtained in the same ultrasound examination may further improve the sensitivity and specificity, and possibly also allow prediction of response based on pre-treatment data [63].

## 5. Evaluation of Intravascular Biomarkers for Angiogenesis Using Targeted Microbubbles

Detection of microbubbles in ultrasound imaging is complex, depending on both biological (target expression) and physical (imaging protocol, microbubble characteristics) parameters. For evaluation of the abovementioned intravascular markers as targets for ultrasound contrast agents, direct comparisons in the same disease models using the same experimental conditions can nevertheless be valuable. As an example, microbubbles targeting VEGFR2 and α_v_β_3_ have been studied in subcutaneous squamous cell carcinoma xenografts, where VEGFR2-targeted microbubbles were found to give 1.6-fold higher signal intensity [32]. Similar findings have been reported in an ovarian cancer xenograft model, in a study of dual VEGFR- and α_v_β_3_-targeting microbubbles. Here, microbubbles targeting VEGFR alone were found to give higher signal intensity than microbubbles targeting α_v_β_3_ alone. Dual-targeted microbubbles were superior to both the two single-targeted microbubbles [37]. However, a longitudinal study of antibody-based microbubbles targeting α_v_β_3_, VEGFR2 or endoglin in xenograft models of breast, pancreatic and ovarian cancer did not show any difference in the performance of α_v_β_3_ and VEGFR2-targeted microbubbles in any model at any time point [34]. In addition to addressing the relative imageability of three potentially useful angiogenic markers, this study demonstrated the importance of experimental model characterization. The signal intensity after administration of endoglin-targeting microbubbles decreased with tumor size, which may reflect both reduced endoglin expression and a relative decrease in tumor vascularity, which is a typical feature of experimental subcutaneous tumors. The study also demonstrated that microbubble retention reflect differences in target expression between different cancers. Endoglin-targeting microbubbles were therefore associated with higher signal intensity than α_v_β_3_-targeting microbubbles in ovarian cancer xenografts, whereas this pattern was reversed in pancreatic cancer xenografts. This illustrates the importance of animal model characterisation and biomarker validation during development of targeted contrast agents. If the receptor expression in an animal model is not adequately described during the selection and optimization phases of development, extrapolation of the findings may lead to false conclusions. Finally, selection of the correct ligand has been shown to have great impact on the imaging performance and specificity of targeted microbubbles. This was illustrated by Willmann *et al*. in a study comparing three different ligands for the α_v_β_3_ integrin [14]. In this study, knottin ligands outperformed both an RGD peptide and an anti-α_v_β_3_-antibody. In summary, the findings from preclinical studies comparing microbubbles must be interpreted with caution. In most of the studies, direct comparison of microbubbles has not been the primary objective, and the studies have been designed to assess other aspects of molecular ultrasound imaging. Due to the variability in angiogenic biomarker expression between cancers, no superior target for molecular ultrasound imaging can be identified from these studies. The body of data demonstrates that microbubbles targeting different angiogenic markers reflect the target expression of the biological systems, a feature which can be of great clinical value.

## 6. Considerations for Evaluating Contrast Agent Performance

**Figure 3:**
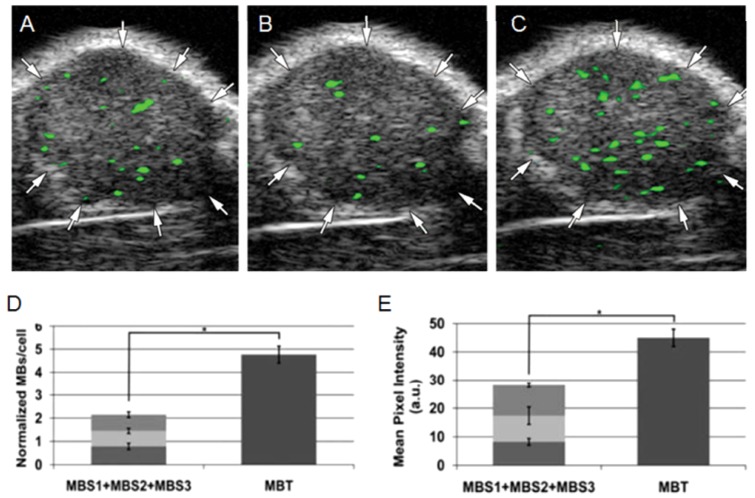
The effect of dual and triple targeting of microbubbles. (**A**,**B**) shows accumulation of microbubbles targeting VEGFR2 or α_v_β_3_, respectively, in a xenograft tumor. (**C**) shows the increased accumulation after injection of microbubbles targeting both VEGFR2 and α_v_β_3_. Binding of triple-targeted microbubbles (*P*-selectin, α_v_β_3_ and VEGFR2) to cells (**D**) and accumulation in xenografts (**E**) has been proven higher than the sum of corresponding amount of single-targeted microbubbles. Reproduced with permission from [37,38].

The studies reviewed in this paper show that microbubbles targeting intravascular disease markers consistently give several-fold higher signal intensity than isotype control microbubbles. The difference between targeted and control microbubbles is in the same order of magnitude for most intravascular targets. Microbubble retention consistently reflects the expression of the target molecule across all studies. The *in vivo* performance of molecularly targeted contrast agents should not be compared without taking the properties of the model systems into account. The expression of target molecules related to angiogenesis is known to differ between models. Even within the same model, target expression can vary with time due to tumor growth or disease progression. There are also several factors related to the microbubble that can affect the target-to-background achieved *in vivo*. For example, the size of the microbubbles may affect the signal intensity that can be achieved. Therefore, it is important to compare bubbles of similar size when evaluating different target molecules [64]. Other important factors are the wall shear rate and the ligand density of the microbubbles. It has been shown that adhesion of microbubbles increase with ligand density, and comparison of different microbubbles must therefore take the ligand density into account [65]. For comparison of imaging performance, contrast agent candidates must therefore be tested in the same animal models under the same experimental conditions, as discussed in section 6. It is, however, becoming increasingly clear that microbubbles with dual or triple targeting are superior to microbubbles carrying only one ligand. Multiple targeting facilitates multiple bindings between the bubbles and the endothelium and increases the adhesion strength to the endothelium and the retention of microbubbles in the tissue (Figure 3). It has been shown that combined targeting of VEGFR2 and α_v_β_3_ is advantageous compared to microbubbles targeting only one of these targets [37]. Furthermore, triple-targeting microbubbles (*P*-selectin, VEGFR2 and α_v_β_3_) have been demonstrated to have even higher tumor retention than dual-targeting microbubbles [38]. Interestingly, sequential administration of single-, dual- and triple-targeted microbubbles in the same individual animals has demonstrated significant synergy effects of multiple targeting. Triple-targeted microbubbles gave approximately 4-fold higher video intensity than any single-targeted microbubble, and 40% higher signal intensity than dual-targeted microbubbles [38].

## 7. Summary and Outlook

The studies summarized above demonstrate that the intravascular compartment in cancer contains several targets that can be imaged using contrast-enhanced ultrasound, for diagnostic or therapy monitoring purposes. For successful development of a targeted ultrasound particle, several aspects must be taken into account. Firstly, the microbubbles must be biocompatible and have physical/acoustic properties matching the intended use. Secondly, a relevant molecular target (or several targets) must be identified. Thirdly, the microbubbles must be decorated with ligands binding to this target with sufficient specificity and affinity, and at a sufficient density, to facilitate binding to the target *in vivo*. Finally, the prototype microbubbles must be evaluated in clinically relevant and well-characterized preclinical models. Most importantly, the targeted microbubbles must produce clinically relevant information that has incremental diagnostic value compared to other diagnostic procedures.

Based on the research summarized in this review, no single molecular target appears to be superior for ultrasound imaging of cancer. In terms of clinical translation, VEGFR2-targeting microbubbles are in early-phase clinical trials and may be the first to reach clinical use. Dual- or triple-targeting microbubbles have repeatedly been shown to outperform single-ligand microbubbles. It is therefore possible that future research will identify combinations of ligands which optimize the imaging performance and allow tailoring of contrast agents for specific purposes. A multi-purpose microbubble decorated with ligands both for angiogenesis and inflammation is an exciting possibility, which could reduce the relative development cost, as the same microbubble product can be used in different indications.

In conclusion, molecular contrast-enhanced ultrasound is well established as a method for functional studies of diseases involving vascular pathology. This has been demonstrated in cancer, where imaging of angiogenic markers with targeted microbubbles have been evaluated both for diagnostic imaging and for monitoring response to antiangiogenic treatment. Progress in ligand and conjugation chemistry has led to the development of contrast agents with clinically desirable properties, and clinical trials have been initiated. Future optimization of ligands and microbubbles may lead to contrast agents that are valuable in management of cancer. We believe that identification of applications which utilize the inherent strengths of ultrasound imaging will be crucial for the introduction of molecular contrast-enhanced ultrasound in the clinic.

## Figures and Tables

**Table 1:** Summary of studies using targeted microbubbles (MBs) to assess tumor angiogenesis.

Target	Ligand	Model System	Tumor Contrast Enhancement Compared to Non-Targeted Control MBs 1	Other Findings	Reference
VEGFR2/KDR	Heterodimeric peptide (BR55)	Mouse Colon carcinoma xenograft LS174T	3-fold	Video intensity corresponds to MVD and VEGFR2 expression, allowing monitoring of antiangiogenic therapy	[26]
VEGFR2	Heterodimeric peptide (BR55)	Rat Mammary carcinoma 13762 MAT B III	NA	The binding specificity of microbubbles with heterodimeric peptide ligand was similar to that of microbubbles with anti-VEGFR2 antibodies	[18]
VEGFR2	Heterodimeric peptide (BR55)	Mouse Breast cancer xenograft MCF-7/MDA-MB-231	NA	2-fold difference in VEGFR2 expression between tumor models reflected in video intensity	[27]
VEGFR2	Heterodimeric peptide (BR55)	Rat Prostate adenocarcinoma G Dunning R-3327	NA	20-fold difference in signal intensity between prostate cancer and normal tissue. Binding similar to that of antibody-coated bubbles	[28]
VEGFR2	Antibody	Mouse Breast cancer xenograft NR67	2.5-fold	Retention of VEGFR2-targeting bubbles correlate to VEGFR2 expression but not vascularity	[29]
VEGFR2	Antibody	Mouse Pancreatic cancer xenograft MiaPaCa2/Pan02	1.5-fold	Reduced endothelial expression of VEGFR after treatment with gemcitabine	[30]
VEGFR2	Antibody	Mouse/Rat Angiosarcoma (SVR) Glioma (C6)	3–5 fold	Unspecific control MBs had significantly higher video intensity than unlabeled MBs (10-fold)	[31]
VEGFR2	Antibody	Mouse Squamous cell carcinoma HaCaT-ras-A-5RT3	7.5-fold	Reduced microbubble retention after matrix metalloproteinase inhibition. No significant difference between VEGFR2- and α_v_β_3_-targeted microbubbles	[32]
α_v_β_3_	Echistatin	Rat Glioma xenograft U87MG	3-fold	Spatial variation in signal intensity corresponded to integrin expression	[33]
α_v_β_3_	Knottin	Mouse Ovarian cancer xenograft SK-OV-3	3-fold	Knottin-decorated MBs outperformed MBs conjugated with RGD or antibodies and had a 12-fold tumor-muscle ratio	[14]
α_v_β_3_	Cyclic RGD peptide	Mouse Breast cancer xenograft Met-1	8-fold		[9]
α_v_β_3_	RGD	Mouse Squamous cell carcinoma HaCaT-ras-A-5RT3	5-fold		[32]
Endoglin (CD105)	Antibody	Mouse Pancreatic cancer xenograft MiaPaCa2	1.5-fold		[30]
VEGFR2 α_v_β_3_ Endoglin (CD105)	Antibodies	Mouse Subcutaneous xenografts MDA-MB361 (breast) SKOV-3 (ovarian) MiaPaCa2 (pancreatic)	NA	Microbubbles targeting endoglin had up to 3-fold higher video intensity than microbubbles targeting VEGFR2 or α_v_β_3_. In pancreatic tumors, microbubbles targeting α_v_β_3_ had the highest video intensity	[34]
Unknown	RRL	Mouse Prostate cancer xenograft PC-3	3-fold	Spatial variation in signal intensity corresponded to vascular density	[35]
ICAM-1 α_v_β_3_	Antibody	Rat Prostate cancer xenograft AT-1	3-fold	Approximately 1.5-fold higher video intensity than RGD-labeled MBs	[36]
VEGFR2 + α_v_β_3_	2 × antibody	Mouse Ovarian cancer xenograft SK-OV-3	4-fold (VEGFR2) 3-fold (α_v_β_3_) 6-fold (VEGFR2 + α_v_β_3_)	Dual-targeted microbubbles outperformed microbubbles with only one ligand	[37]
VEGFR2 + α_v_β_3_ + ICAM1	3 × antibody	Mouse MDA-MB-231	NA	Triple-targeted microbubbles had 1.6-fold higher signal intensity than the additive intensity of all three single-targeted microbubbles, and approximately 5-fold higher VI than any of the single-targeted microbubbles alone	[38]

1 The figures are not directly comparable, as different disease models express varying levels of angiogenic markers, and the imaging parameters and time points are varying. Data are either obtained from quantitative tabular data or visual interpretation of graphical representation of imaging performance in the reviewed papers.
